# The Health and Well-Being of Women and Girls Who Are Refugees: A Case for Action

**DOI:** 10.3390/ijerph22020204

**Published:** 2025-01-31

**Authors:** Jinane Chalouhi, David C. Currow, Nuhad Yazbik Dumit, Shailendra Sawleshwarkar, Nancy Glass, Sophie Stanfield, Michelle Digiacomo, Patricia M. Davidson

**Affiliations:** 1 Previous Vice Chancellors Unit, University of Wollongong, Wollongong, NSW 2522, Australia; drjchalouhi@gmail.com; 2 Faculty of Health, University of Technology Sydney, Ultimo, NSW 2007, Australia; david.currow@uts.edu.au (D.C.C.); michelle.digiacomo@uts.edu.au (M.D.); 3 School of Nursing, American University of Beirut, Beirut P.O. Box 11-0236, Lebanon; ny00@aub.edu.lb; 4 Faculty of Medicine and Health, University of Sydney, Sydney, NSW 2006, Australia; shailendra.sawleshwarkar@sydney.edu.au; 5 School of Nursing, Johns Hopkins University, Baltimore, MD 21205, USA; nglass1@jhu.edu; 6 Graduate School of Medicine, University of Wollongong, Wollongong, NSW 2522, Australia; sophie.stanfield@icloud.com; 7 Vice Chancellor’s Unit, University of New South Wales, Sydney, NSW 2033, Australia

**Keywords:** women, girls, refugees, social determinants of health, intersectionality, gender-based violence, discrimination, racism, migration

## Abstract

The plight of displaced people is an escalating global challenge. No longer solely the domain of individuals working in humanitarian settings, the plight of refugees is now a very visible aspect in mainstream health and social services. Refugee women and girls face serious and interconnected health challenges due to forced displacement, limited access to healthcare, gender-based violence, exploitation, and other factors affecting their health and well-being, particularly social determinants of health. These experiences are often built upon intergenerational forms of abuse such as enduring colonial and patriarchal models where there are fundamental power imbalances and impediments to economic and political stability and as a consequence health and well-being. One in five displaced women and girls experiences sexual violence, which has lasting effects on their physical and mental health. Moreover, financial instability and uncertainty in migration status can further push women and girls into exploitative circumstances, such as modern slavery and survival sex. This paper presents a scoping review using a gender-based lens aimed at analyzing the social determinants impacting the health and well-being of refugee women and girls. The environmental, socio-economic, cultural, and gender-specific drivers of security are described. Advocating for strategies to promote health equity, protection, resilience, and empowerment for refugee women and girls is important for their health and well-being. Achieving this is critical in contributing towards building stronger, healthier, and resilient communities, and creating a buffer to the escalating numbers of people being driven from their homes worldwide.

## 1. Introduction

One in every sixty-nine people, or 1.5 per cent of the world’s population, is now displaced from their place of birth, either fleeing internally or to another country. Of the 117 million individuals forcibly displaced, 50% are women and 40% are children [1]. Geopolitical conflicts, climate effects, human rights abuses, and economic deprivation are key drivers for these demographic shifts [2]. This trend is likely to continue due to increasing political conflict and extreme weather events internationally [3]. War and conflict do not just maim and kill but the disruption provides a substrate for infection, disease, and corruption that is often passed across generations.

The world has become less peaceful over the past two decades, with conflict widely distributed globally. The Institute for Economics and Peace outlines a deteriorating situation. There are 56 current active conflicts, the most since the end of World War II. The Syrian conflict is one of the largest crises in the world and these refugees have sought asylum in more than 130 countries, with the vast majority living in neighboring countries, such as Türkiye, Lebanon, Jordan, Iraq, and Egypt, all of whom are experiencing resource shortages and political upheaval. Most Syrian refugees rely on humanitarian assistance to survive. The conflict in Gaza, Syria, Ukraine, and Sudan also illustrate multiple flashpoints around the world and pressures placed on host countries to care for refugees [4,5]. Once predominantly the remit of specialized agencies, such as the United Nations High Commission for Refugees (UNHCR), the care of refugees is now part of mainstream healthcare, where there is limited experience and capacity often situated within highly political and contested settings. Countries, such as Lebanon, struggling socially, politically, and economically, are home to many refugees, further exacerbated by the escalation of conflict in the Middle East [6].

Recasting a focus on refugee health is important in addressing the challenges of contemporary society as well as the healthcare ecosystem. This involves recognizing the unique needs of refugee populations and a focus on sex- and gender-based differences, specifically the needs of women and girls. The increasing prevalence of displaced populations underscores the importance of mainstream health services being engaged in refugee health and therefore obtaining a deeper understanding of their experiences and needs. The increasing recognition of intergenerational trauma means that the refugee experience cannot be confined to a single point in time. It is highly likely that for many individuals, the experience of displacement and refugee status is a defining adverse life experience endured across the lifespan and potentially across future generations [7].

The exposure of refugees to adverse social, health, and economic consequences is undeniable, but for women and girls, the vulnerability and potential for adverse events is amplified [8,9]. Sexual violence is often used as a weapon of war, exacerbating the adverse impacts of the disempowerment of women in some societies. Addressing social determinants of health, and, in particular, promoting gender equity, are recognized as fundamental elements of achieving the United Nations (UN) Sustainable Development Goals (SDGs) [10]. In order to address these issues, we need to consider specific definitions, as the nomenclature has serious implications under international law and resettlement conditions.

A refugee is a person who has fled their own country because they are at risk of serious human rights violations and/or persecution. Refugees have a right to international protection. The 1951 Refugee Convention and its 1967 Protocol governs the movement of refugees and asylum seekers across international borders. There is increasing criticism of the utility of this framework given changing views of territoriality in a globalized world [11]. Internally displaced people (IDP) are those who have been forced to flee their homes by conflict, violence, persecution, or disasters, remaining within the borders of their own country and not crossing national borders. The 2024 Global Report on Internal Displacement details the scale and impacts of internal displacement. The total number of people living in internal displacement has increased by 51% over the past five years, reaching a record high of 75.9 million people across 116 countries at the end of 2023 [12].

Seeking asylum is a human right. An asylum seeker is a person who has left their country and is seeking protection from persecution or serious human rights violations in another country, but who has not yet been legally recognized as a refugee and is waiting to receive a decision on their asylum claim. Many refugees remain displaced for many years, and the number of refugees under UNHCR’s mandate reached 37.4 million at the end of 2023, 2.7 million more than at the end of 2022. To ensure that the needs of refugee women’s and girls’ health and well-being are promoted, it is important to understand their experiences and develop solutions within their world views, the complexity of their lives, and the systems and structures in which they live.

The UNHCR has increased the focus on gender equality, particularly focusing on sexual and gender-based violence. At the 2023 Global Refugee Forum, the Poverty Alleviation Coalition committed to initiate actions utilizing the Graduation Approach, aiming toward poverty relief targeting 190,000 households impacted by displacement and host communities across 35 countries in Africa, Asia, Europe, Latin America, and the Middle East from 2024 to 2027. Following extensive consultation, the WHO has launched a guide to focus research efforts to address the health needs of migrants, refugees, and all forcibly displaced populations, and shape responsive policies and practices worldwide [13].

The purpose of this manuscript was to synthesize existing data identifying factors contributing to the health needs of refugee women and girls, shedding light on the sex and gender dimensions of forced displacement, and recommending targeted responses. This approach is necessary due to the rapidly escalating refugee crisis, disparate information sources, the increasing importance of refugee health in mainstream health services, and the need for gender-specific approaches.

In this paper, we asked the question, what are the barriers and facilitators to promoting the health and well-being of refugee women and girls at the individual, interpersonal (micro), organizational, community (meso), and societal (macro) levels?

## 2. Materials and Methods

**Figure 1:**
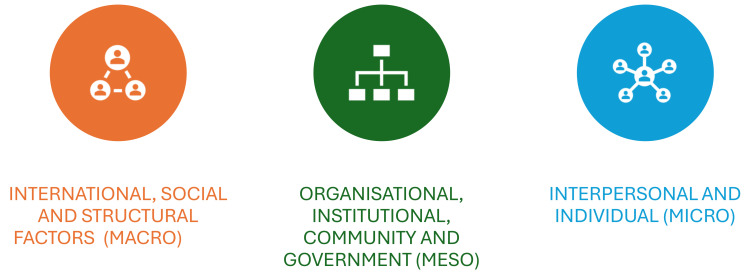
Social ecological framework.

A scoping review was identified as the most effective way to undertake this review for the following reasons: (1) the rapid changes in the refugee status and volatile global situation; (2) the volume of information in the gray literature; and (3) the need to explore different concepts embedded in structural frameworks. In addition to using the Social Ecological Model (SEM) as an overarching framework, this approach provides an inclusive approach to understanding the dynamic, multifaceted, and inter-related factors influencing not only the circumstance of leaving their home country but also settlement and adjustment. These multilevel factors range from societal (macro), organizational, and community (meso) to individual (micro) levels (Figure 1) [14,15].

Social determinants of health (SDHs) were considered important in synthesizing data due to their impact on health and influence on the migration experience [16]. Social determinants of health are the circumstances in which people are born, grow, work, live, age, and die. These manifest in the economic policies and systems, social norms, social policies, and political systems that shape the lives of women and girls. The concept of intersectionality was also applied to understanding the complex, cumulative ways in which multiple elements of discrimination, such as racism, sexism, and classism, interact and intersect to, in some instances, create cumulative and longitudinal disadvantage. This approach considers attributes such as sex, race, and class as intersecting identities that shape experiences [17].

A search of the literature published between January 2014 to July 2024 was conducted in six databases (Scopus, Medline, CINAHL, PsychInfo, Pubmed, and Web of Science). Using a Boolean search strategy and MesH terms, key words included “refugee* status” AND “women* OR “girl*”, “healthcare services”, “conflict”, and “human rights”. Reference lists were scanned to ensure all related publications were included. No exclusion criteria were applied regarding methodology or study setting. All study designs were integrated in the review, including commentaries and opinions. The Google search engine was used to identify relevant policy documents and gray literature. The search was restricted to articles published in English. Identifying and interpreting data from the research review data from the scoping review involved identifying key themes using textual analysis, which allowed the categorization of findings. Data were managed in Endnote 21. Applying a deductive approach, the social determinants of health, the social ecological framework, and a position of intersectionality were used as frameworks that guided analysis and synthesis.

## 3. Results

**Figure 2:**
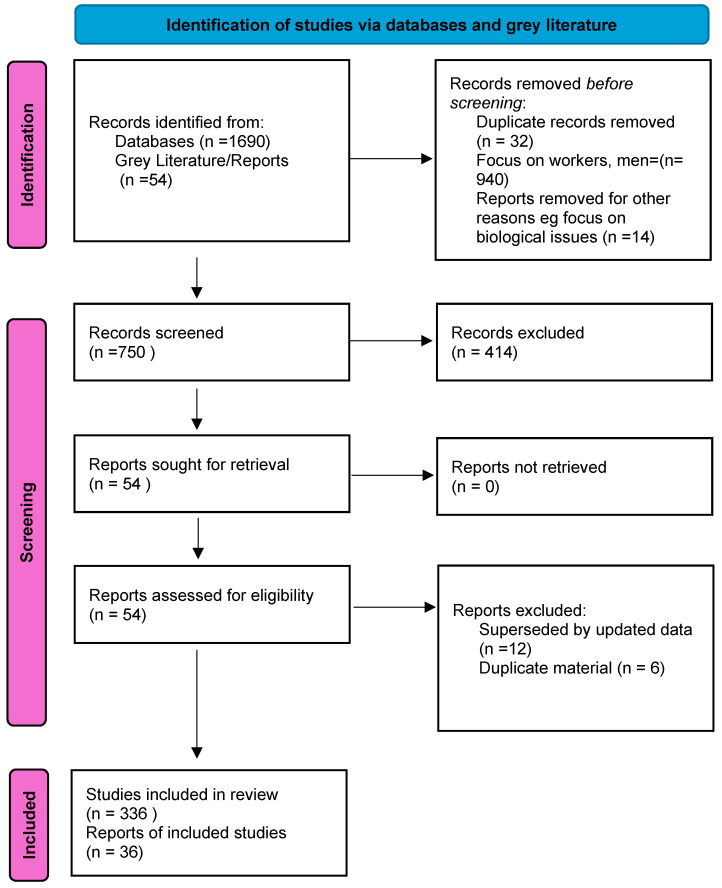
Prisma flowchart.

Using the search strategy, 1690 articles were sourced and 750 articles were identified as relevant to the review, as shown in Figure 2. The abstracts of each of these articles were reviewed and key texts retrieved pertaining to the review question and relevance to social determinants of health and the social ecological framework. Full-text reports from peak bodies and non-government organizations provided access to contemporaneous statistics. Relevant data were extracted from documents from key organizations such as the UNHCR and the WHO. The increasing significance of refugee health is reflected in the increasing numbers of studies and commentaries in the literature. For example, in PubMed in 2003 there were 244 articles, whereas in 2022 there were 1690. The findings of the review strategy were organized around the following headings:

1. Scope of the problem and barriers and facilitators to promoting health and well-being and/or resettlement.
2. Framing of the problem using the lens of social determinants of health, the social ecological framework, and the application of principles of intersectionality.
3. Identification of high-risk situations and vulnerability.
4. Pillars for action to improve the health and well-being of women and girls who are refugees based upon assessment of barriers and facilitators to settlement.

### 3.1. Scope of the Problem

The health of refugee women and girls has been shaped by several interconnected factors at the macro, meso, and micro levels. These factors interact to shape the overall health experiences of refugee women, highlighting the need for a multifaceted, comprehensive approach to addressing their health and social needs. Currently, there are at least 36.4 million people classified as refugees under the protection of the UNHCR, having crossed international borders [18]. Of these, 6.1 million people are asylum seekers, seeking international protection in another country while awaiting decisions on their refugee status. The asylum process can be long and complex, leaving many in a state of legal uncertainty and vulnerability over many years or sometimes decades. Of these, 5.3 million individuals are under international protection, including those granted asylum or other forms of temporary protection. They are either individuals facing risks if returned home but who do not meet refugee criteria or, less often, individuals offered immediate refugee status, such as those fleeing armed conflict or environmental disasters [19]. The majority of the literature yielded as part of the search strategy described issues pertaining to mental health, gender-based violence, and reproductive health [20]. The barriers and facilitators to access to healthcare for women and girls who are refugees are summarized in Table 1 [20].

### 3.2. Framing of the Problem

Using the organizing framework, issues pertaining to macro, meso, and micro factors are summarized below.

#### 3.2.1. Macro: International, Social, and Structural Factors

At the macro level, there are a number of policies and legislative and normative issues that influence the experiences of refugees. A range of international laws and mandates of peak bodies such as the United Nations and World Health Organization influence the motivation and actions of individual nation states and international policies on refugee rights [21]. Social factors contributing to the experience of women and girls included racism, stigma, discrimination, and policy perspectives on gender, ethnicity, and culture [22].

#### 3.2.2. Meso: Organizational, Institutional, Community, and Government

Policies of specific countries, healthcare organizations, and the availability of resources, impact the access of women and girls to healthcare services and quality care [23]. Due to previous disadvantage, the migration journey, and often traumatizing experiences, refugee women and girls require unique healthcare programs including the following: mental health support; access to social services; reproductive health services and family planning; transport assistance; and language interpretation services [24]. At this meso level, localized influences such as community acceptability, cohesion and support, and the availability of culturally sensitive local healthcare systems influence experiences. Experiences across a range of countries vary, but issues remain remarkably similar. In countries without universal healthcare coverage, the cost of care is a barrier to access.

Inadequate communication, racism, and stigma in the host community and power imbalances are issues that are widely experienced [25,26]. Refugees settled in regional areas often face additional challenges. These include competition between the host community and refugees over scare resources [27,28]. Local non-government organizations and community support networks often play crucial roles in providing a safety net and support in facilitating access to essential health services [29]. Yet, the absence of legislative frameworks can often hinder resource allocation and policy formulation [27].

#### 3.2.3. Meso: Interpersonal

Displaced from their homelands, refugee women and girls often rely heavily on the local social support networks available to them in the host country through both formal and informal means. Refugees often struggle with social isolation and increased levels of stress, anxiety, depression, and loneliness due to a variety of factors like family separation, language barriers, past traumas, an unstable immigration situation, housing instability, lack of employment, discrimination, and unfamiliarity with the host country’s culture [30,31]. Many refugees may also grapple with preserving their traditions and values [32]. Community cohesion and interpersonal relationships within their communities and families can provide a strong sense of belonging, safety, and a modest financial backing, positively affecting their emotional well-being and adjustment struggles [33].

#### 3.2.4. Micro: Individual

Refugees are often the most vulnerable individuals in society, suffering considerable disadvantages and persecution before fleeing their home countries. These adverse circumstances are transposed and aggravated on relocation [34]. Housing stability, level of education, language skills, past trauma, and mental health issues influence the refugee’s experience [16]. Refugee women and girls come from diverse cultural and religious backgrounds with specific health-seeking beliefs and preferences. Healthcare providers in host countries must respect these differences and focus on establishing trust. Systemic racism and discrimination adversely impact health outcomes [35]. This is a critical issue in the vulnerability of refugee women and girls as they struggle with exploitation and abuse, leading to trauma and stress, highlighting the crucial need for culturally sensitive and appropriate healthcare interventions. The literature to date strongly emphasizes mental and reproductive health and has a lesser focus on non-communicable diseases, which can affect health outcomes [36].

Within the conceptualization of social determinants of health, exposure to racism, alienation, marginalization, and stigma should be considered in planning models of intervention and support [37]. Adopting a lens of intersectionality can also assist in appreciating the impact of being a refugee as well as appreciating structural elements that conspire to potentiate disadvantage [38]. In particular, intersectionality recognizes the diversity and dynamism of the individual’s experiences, which are often context-dependent and demonstrate how these identities intersect to create cumulative disadvantage. For example, the coexistence of racism, poverty, and sexual discrimination can impact employment opportunities [39].

### 3.3. High-Risk Situations and Situations of Vulnerability for Women and Girls

Traditional models of women’s health have had a primary focus on reproductive health, reflecting patriarchal and post-colonial structures, and an emphasis on addressing maternal and infant mortality. This model often fails to address the broader health concerns for women and girls across the lifespan, particularly non-communicable diseases. Invisibility, fear, vulnerability, and uncertainty commonly shape the refugee story of women and girls. Identifying crucial areas for focus and attention is important, particularly for women who are younger, older, or living with a disability or with increased vulnerability, such as being forced into sex work [40,41,42]. Women in humanitarian settings are often highly vulnerable. In addition to patriarchal norms enabling sexual abuse and feelings of shame and stigma, many women are highly dependent on these services. A perspective that sexual relations are ‘part of the deal’ may lead to beliefs that objections will not be effective due to entrenched power imbalances [43]. Women and girls are also subject to modern slavery, where an individual exerts power and control over another. Examples of this are forced marriage, trafficking, and debt bondage [44,45].

#### 3.3.1. Gender-Based Violence and Exploitation

Sexual and gender-based violence is the manifestation of power inequalities between women and men. In areas of conflict, rape is recognized and frequently used as a weapon of war. One in five displaced women and girls have endured sexual violence [46,47]. Different forms of sexual violence (rape, sexual assault, exploitation, and trafficking) often occur in displacement settings, during the migration journey, in refugee camps, and detention centers, where protection mechanisms and safeguarding measures are frequently weak or non-existent. Victims often endure long-lasting repercussions affecting their mental health and exacerbated by their fear of reporting the abuse, normalizing the abuse, and the associated stigma [48].

Overcrowded and poorly managed refugee camps often lack secure shelters and private sanitation facilities, creating conditions where women are more susceptible to attacks. Additionally, financial instability and a lack of basic needs, resources, and economic opportunities can force displaced women into exploitative situations, including survival sex, where they exchange sex for food, shelter, and sometimes protection [42]. Moreover, some cultural and societal norms devalue women and girls and normalize gender-based violence. The trauma inflicted by gender-based violence has long-lasting effects on women’s physical and mental health, including sexually transmitted infections, physical injuries and psychological traumas, anxiety, depression, and post-traumatic stress disorder. Period (menstrual) poverty and limited access to supplies in camps is a source of distress and concern for many women and girls. This distress in also manifested in increased depression, anxiety, and stress [49].

#### 3.3.2. Women and Girls Who Are Refugees with a Disability

An estimated 1.3 billion people, about 16% of the global population, experience significant disability, and this proportion is represented in individuals who are forcibly displaced [50]. In 2019, an estimated that 12 million of the nearly 80 million people forcibly displaced globally at that time were persons with disabilities. Individuals with a disability are often invisible in the health discourse and, as a consequence, there has been less discussion and attention. A recent report has called upon all WHO Member States to take action to advance health equity for persons with disabilities [50]. A range of disabilities, both physical and mental, increase the vulnerability of women and girls who are refugees [51].

Refugee women and girls with disabilities encounter serious discrimination, including isolation and neglect in their daily lives. The key challenges facing refugee women with a disability is the lack of prompt and accurate assessment and support whether on arrival or once settled [52]. Discourses of ableism and ageism emphasize the importance of considering people’s experiences with the lens of intersectionality [53]. There is also the important question of the protection of individuals who need augmented support, particularly in areas such as reproductive health.

Achieving successful integration for refugee women with disabilities relies on recognizing and eliminating existing barriers to ensure equitable access to services and full participation in the host community. Neglecting to address these barriers [54] during the integration phase is the root cause of generating more discrimination and fostering deeper isolation. Available studies and opinions emphasize approaches that embrace diversity and promote a resilience framework [53,54]. The WHO has determined that there is a USD 10 return per USD 1 spent on implementing disability-inclusive prevention and care for non-communicable diseases. Other population-wide interventions such as family planning and vaccination also remain highly cost-effective when provided in a disability-inclusive manner, despite the additional cost required [55].

#### 3.3.3. Older Refugee Women

Older migrants comprise approximately 12% of the global migrant population and the majority of these individuals are women. These individuals commonly experience inferior health status and have numerous healthcare needs [56]. Older women refugees are particularly vulnerable due to age-related issues, disabilities, limited mobility, financial insecurity, and feelings of isolation and loneliness due to disrupted familial and social ties. Moreover, fragile older women refugees are susceptible to exploitation and gender-based violence. Although it is reported that the disease burden is similar between men and women, there is limited discussion of both sex- and gender-based differences [57].

Beyond a deficit narrative, it is important to recognize that older refugees frequently serve as custodians of their community’s cultural heritage; they foster intergenerational cohesion and provide cultural wisdom to the community and younger generations. This role holds particular significance for refugee children and adolescents, due to their ability to support connections to cultural origins and values [58]. Many older adults play a critical role in child care, particularly in large and extended families.

#### 3.3.4. Women and Girls Who Identify as LGBTIQA+ Refugees

Individuals who identify as LGBTIQA+ often face marginalization and discrimination due to their sexual orientation or gender identity, as well as other intersecting factors such as race, ethnicity, religion, and socioeconomic status [59,60]. Although this is increasingly recognized within a human rights framework, stigma and reticence in applications for asylum remain [61]. LGBTIQA+ people often do not receive support from their families and, in some instances, have been subject to abuse [62].

### 3.4. Pillars for Action to Promote Refugee Women’s and Girls’ Health

The extant literature has helped to identify the gaps where further research is needed and implications for promoting new targeted initiatives and policies. Moreover, it has also identified elements of resilience and assets of refugee women and girls. Multilevel solutions at the individual, community, and national government level are required, as well as multisector collaboration engaging civil society, the private sector, and the government. Developing novel approaches is critical for improving the health and quality of life of women and girls who are refugees.

Taking into account the importance of social determinants of health and the social ecological framework, sustainable solutions to improve the health of women and girl refugees require a multilevel approach, starting by addressing immediate healthcare needs and establishing long-term strategies for health, well-being, and resilience. There is a need to understand the specific needs and vulnerabilities of high-risk groups such as older women and young girls as well as their strengths and assets.

Effective partnerships are crucial, requiring effective collaboration between international, national, government, and non-government organizations to tackle discrimination, gender-based violence, and exploitation. Administrative processes and partnerships among various community organizations need to be more efficient; policies should be streamlined, along with proactive advocacy for policy changes. Establishing community support networks, engaging both the refugee and host communities’ involvement, as well as economic empowerment by building skills will also play a significant role in enhancing the integration process.

**Figure 3:**
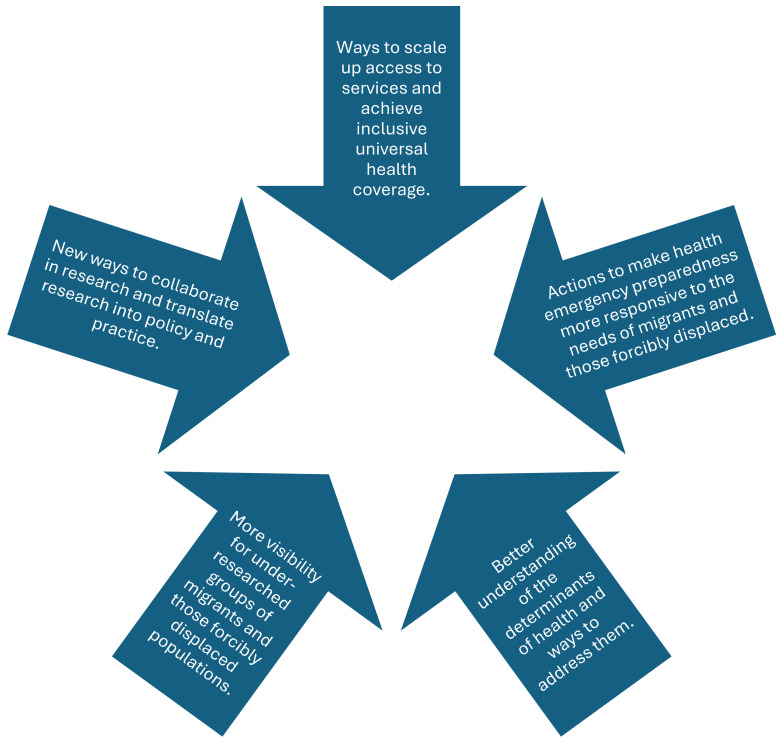
Addressing the rights and needs of refugees, internally displaced persons, and stateless individuals.

Addressing the rights and needs of refugees, internally displaced persons, and stateless individuals is integral to achieving the SDGs. The key recommendations for addressing the needs of women and girls who are refugees are provided in Figure 3.

## 4. Discussion

The findings of this review demonstrate that the numbers of women and girls who are refugees is increasing, and they face a range of challenges and their assets and skills are commonly not recognized. The majority of the existing literature focuses on the immediate concerns of women and girls, in particular, on reproductive and mental health and much less on non-communicable diseases. Across geographical boundaries, there is marked similarity in experiences, views, and aspirations [40,63,64]. Given the protracted conflicts and crises globally, there is an increasing need to embed the refugee agenda within a broader health and social care agenda. To tackle these challenges, comprehensive whole-of-society approaches, encompassing healthcare, partnerships, and integration, are needed. Addressing barriers such as limited education, lack of financial support, poor living conditions, and geographic obstacles to accessing healthcare is crucial [65]. Additionally, cultural and language barriers, gaps in health literacy, gender-based violence, and mental health stigma must be overcome, and a comprehensive, gender-focused approach should be applied across the lifespan.

Effective partnerships are critical and require enhanced collaboration among national, international, government, private, and non-governmental organizations. Simplifying administrative processes and improving cooperation among community organizations are necessary, along with advocating for policy changes to combat trafficking.

For successful integration, building skills, eliminating stigma and discrimination, and fostering community involvement are key. Fostering peer support networks, promoting economic empowerment, and encouraging cross-cultural exchange will greatly improve the integration process.

A recent Statista survey revealed that while 74% of individuals believed that other countries should be able to take refugees avoiding persecution and war, 43% felt that their borders should be closed because their country was saturated in capacity [66]. While nationalism and populism gain in strength across the globe, addressing misinformation and disinformation becomes increasingly important [67,68,69]. All too often, migration is a political football, with strategies addressing short-term political expediency rather than longer-term strategies addressing both human rights and economic productivity.

### 4.1. A Resilience Narrative—Positive Impact of Refugee Women to the Host Countries

**Figure 4:**
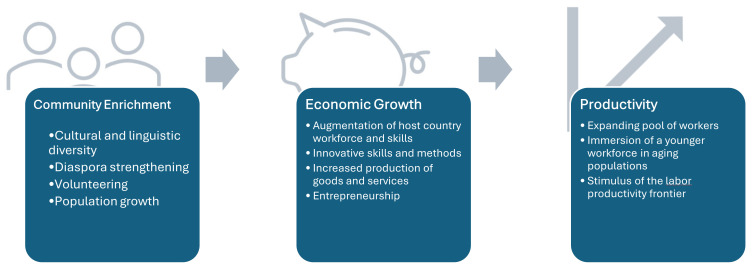
Benefits of engaging refugee women and girls in society.

The common narrative surrounding refugees reflects a deficit model where failings are attributed to an individual rather than the system. But, there are many opportunities and case studies demonstrating the contributions and successes of refugees when they are accepted and valued [70]. Refugees bring significant benefits to their new country, despite the challenging and costly initial resettlement and adjustment costs. Once settled in their new environment, refugees quickly make remarkable cultural and social impacts, adding multiculturalism to their communities and dynamic economic growth. It is estimated that refugee women could contribute an additional USD 1.4 trillion to the annual global gross domestic profit if provided working rights and opportunities [71]. Currently, 70 percent of refugees live in countries that restrict their right to work yet face workforce shortages. Empowering refugees to earn a living is critical if they are to rebuild their lives and benefit the communities they live in. Many refugees, when given the right to work, launch their own businesses, supporting their families and boosting local economies [72]. Figure 4 outlines the potential benefits to society of the engagement of women who are refugees in the economy.

Ensuring the health and well-being of refugee women and girls is both a moral imperative and a crucial aspect of achieving the SDGs. These women and girls face distinct health challenges due to forced displacement, limited healthcare access, and gender-based violence. Multiple social determinants, such as poverty, housing, education, employment, and social support, profoundly impact the health of refugee women and girls [73,74].

Addressing these factors requires a comprehensive approach to policy development and intervention on a global scale, aiming to reduce health disparities. Understanding the concept of intersectionality, recognizing how factors like ethnicity, gender, and socioeconomic status interact is essential for creating effective, sustainable, and refugee-centered solutions. Recognizing these interactions helps in creating more nuanced and effective health policies and interventions that cater to the specific needs of refugee women. This is important in understanding that the experiences of women and girls are unique and cannot be stereotyped [70].

Until recently, there has been a minimal focus on populations at higher risk, such as individuals with a disability [75]. Similarly, women and girls who identify as LGBTQIA+ are at special risk due to discrimination and lack of culturally appropriate services [76]. In some host countries, such as Lebanon, pressure from conservative forces to limit the congregation of LGBTQIA+ individuals has led to further alienation and marginalization, emphasizing the importance of considering social determinants of health and intersectionality [76]. Older refugee women, often seen as custodians of cultural heritage, require specialized healthcare and social support to manage age-related issues and integrate smoothly into host communities [77]. For example, a study in Poland demonstrated that the healthcare needs of older Ukrainian refugees require considerable augmentation of existing infrastructure to meet their needs [78]. Throughout their difficult journeys, refugee women and girls encounter significant legal barriers that impede their access to basic rights and needs. Uncertain visa status can limit access to health and social services [79]. Strengthening legal protections is crucial for safeguarding their rights and well-being, preventing exploitation and discrimination. A number of strategies, such as the Refugees Compact, are binding for many nation states, though the limitations are increasingly recognized. [80,81].

Healthcare access is a precarious issue for refugee women, who often suffer from both physical and mental trauma and chronic health problems due to displacement. A critical issue for women and girls is challenges in accessing sexual and reproductive health services [64]. This may be due to a number of factors, including lack of appropriate education, poor knowledge, attitudes and behaviors, community norms, lack of leadership and trained personnel, language, and socio-cultural barriers [82]. Culturally sensitive healthcare services, including mental health support and reproductive healthcare tailored to their specific needs, are essential. Education is another key component for promoting adjustment. Programs designed to meet the needs of refugee women, including language and vocational skills training, can open up job opportunities and facilitate social integration and entrepreneurship [72]. Economic empowerment, through employment opportunities and tailored training programs, is vital for enhancing their independence and quality of life. Government grants can further help refugee women start their own businesses, achieving financial stability. Universities can also play a critical role in supporting women and, in particular, look to harmonize qualifications [83,84].

Evidence-based policy-making is essential to create effective global strategies and move from short-term utilitarian strategies [85]. Research and data collection provide valuable insights into the needs and challenges faced by refugee women and girls, guiding policy development and program design. Community centers, support groups, and mentorship programs can help provide refugee women and girls with a sense of belonging and facilitate smoother integration into their host communities. These support systems should also be inclusive of disabled and elderly refugee women, ensuring they receive the necessary support and protection.

### 4.2. Pathway for Action

Global circumstances indicate that the number of individuals who are refugees is going to increase with existing conflicts in Palestine, Lebanon Syria, Ukraine, South Sudan, and Gaza and the rising number of climate emergencies globally. Once the remit of specialized services, refugees are now commonly accessing mainstream services, emphasizing the importance of culturally appropriate, safe, and responsive healthcare. This will not occur without the investment and focus of all health professionals, not just humanitarian services.

**Figure 5:**
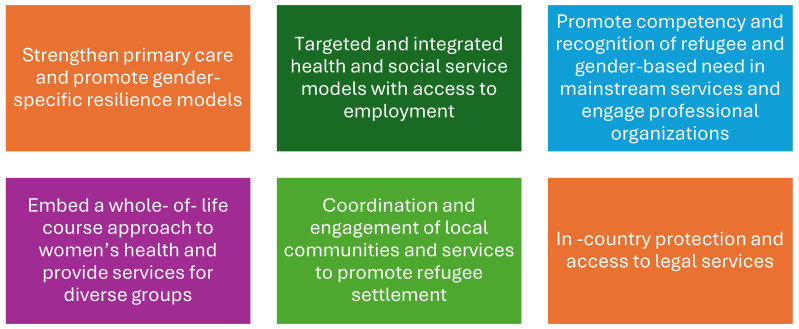
Pillars for action to improve the live of women and girls who are refugees.

There is a legacy in humanitarian services of being reactive, and some have challenged these approaches for the ‘white savior’ foundations, which can potentiate entrenched inequalities and create a substrate for abuse [86]. Importantly, there is a need for ongoing longitudinal studies on the experiences of refugees to inform evidence-based policy [87,88]. To date, the majority of research focuses on the immediate flight period. Given the increasing evidence of the impact of intergenerational trauma, this is an important focus for future research, as well as non-communicable conditions and health across the life course [89]. Research is also important to inform the pre- and post-service training/curricula of healthcare professionals to provide competent healthcare for better health outcomes of refugees, in addition to informing policy [90,91]. Based upon our review, we present in Figure 5 pillars for actions for mainstream refugee health and focus on the needs of women and girls.

This review has the inherent limitations of a scoping review, as it does not have the evidence synthesis of a systematic review. However, the breadth of this review has generated data from a range of sources and domain areas. The synthesis of information has been informed by conceptual frameworks underscoring the intersection of macro, meso, and micro effects and the importance of the social determinants of health. This approach has allowed the generation of key pillars to improve the health and well-being of women and girls. This review has elicited both trends in focus on specific areas as well as identified areas of lesser focus, such as older women and those with disabilities.

## 5. Conclusions

Our world is experiencing unprecedented political, social, and climate upheaval. Escalating conflicts compounded by the economic impacts of disasters are forcing hundreds of millions to flee their homes. Fifty percent of refugees, either stateless or internally displaced, are women and girls. Improving support for refugee women globally requires a comprehensive and inclusive approach including both state- and non-state-based actors. By integrating better healthcare access, robust legal protections, economic empowerment opportunities, educational access, comprehensive social support systems, intersectional awareness, and evidence-based policy-making, we can develop sustainable solutions that uplift and empower refugee women. International cooperation and a commitment to gender-sensitive approaches are essential to ensure that no refugee woman or girl is left behind.

## Figures and Tables

**Table 1:** Barriers and facilitators to healthcare for women and girls who are refugees.

Barriers	Facilitators
Entrenched colonial and patriarchal attitudes Ineffective collaboration between international, national, government, non-government, and private organizations Racism, discrimination, and stigma Poor and abusive living and settlement conditions Gender-based violence Fragile visa status and absence of legal support Trafficking and modern slavery Limited education, health literacy, and access to information Transport and access to health services Lack of access to mainstream health services Cultural, language, religious, and socioeconomic factors Lack of trust in authority Absence of gender-specific and culturally appropriate services Residing in rural and remote areas	Community engagement in resettlement programs A robust primary healthcare system that is accessible and affordable Access to transport, interpreting, and language services Health professionals who are respectful, accessible, and supportive Liaison and coordination with other services, particularly, housing, education, and employment Information and contacts related to asylum services Access to specialized refugee services, including mental health Comprehensive healthcare with access to mental health services Assistance with navigation of the health and social services An integrated and comprehensive approach to physical and mental health

## References

[1] United Nations. Refugee Agency Global Trends. https://www.unhcr.org/global-trends#:~:text=One%20in%20every%2069%20people%2C%20or%201.5%20per,125%20people%20who%20were%20displaced%20a%20decade%20ago.

[2] Lenshie N.E., Ojeh V.N., Oruonye E.D., Ezeibe C., Ajaero C., Nzeadibe T.C., Celestine U.U., Osadebe N. (2022). Geopolitics of climate change-induced conflict and population displacement in West Africa. Local Environ..

[3] Feng Y., Sabir S.A., Quddus A., Wang J., Abbas S. (2024). Do the grey clouds of geopolitical risk and political globalization exacerbate environmental degradation? Evidence from resource-rich countries. Resour. Policy.

[4] Moayerian N., Stephenson M. (2023). UNHCR, National Policies and the Syrian Refugee Crisis in Lebanon and Jordan. https://www.e-ir.info/author/neda-moayerian-and-max-o-stephenson-jr/.

[5] Seita A., Al-Jadba G. (2023). Gaza is facing a humanitarian catastrophe. Lancet.

[6] Fakhry Y., Hassan H.F., Diab J.L. (2024). Health in Crisis: A Paradox of Access for Syrian Refugees and Lebanese Hosts. Health Serv. Insights.

[7] Shoib S., Armiya’u A.Y.U., Swed S., Naskar C., Chandradasa M., Tsagkaris C., Zaidi I., Saeed F. (2024). The importance of addressing intergenerational trauma in refugees. Nat. Ment. Health.

[8] Glass N., Perrin N., Clough A., Desgroppes A., Kaburu F., Melton J., Rink A., Read-Hamilton S., Marsh M. (2018). Evaluating the communities care program: Best practice for rigorous research to evaluate gender based violence prevention and response programs in humanitarian settings. Confl. Health.

[9] Shishehgar S., Gholizadeh L., DiGiacomo M., Davidson P.M. (2023). Arrived, Yet In-between: Experiences of Iranian Asylum-Seeker Women Living with Insecure Residency in Australia. J. Int. Migr. Integr..

[10] Denaro C., Giuffré M. (2022). UN Sustainable Development Goals and the “refugee gap”: Leaving refugees behind?. Refug. Surv. Q..

[11] Benhabib S. (2020). The end of the 1951 refugee convention? Dilemmas of sovereignty, territoriality, and human rights. Jus Cogens.

[12] Internal Displacement Monitoring Center 2024 Report on Global Displacement. https://www.internal-displacement.org/global-report/grid2024/.

[13] World Health Organisation Global research agenda on health, migration and displacement. https://www.who.int/publications/m/item/global-research-agenda-on-health--migration-and-displacement-overview.

[14] Hawkins M., Schmitt M., Adebayo C., Weitzel J., Olukotun O., Christensen A., Ruiz A., Gilman K., Quigley K., Dressel A. (2021). Promoting the health of refugee women: A scoping literature review incorporating the social ecological model. Int. J. Equity Health.

[15] Logie C., Okumu M., Mwima S., Hakiza R., Irungi K., Kyambadde P., Kironde E., Narasimhan M. (2019). Social ecological factors associated with experiencing violence among urban refugee and displaced adolescent girls and young women in informal settlements in Kampala, Uganda: A cross-sectional study. Confl. Health.

[16] Taylor J., Haintz G. (2018). Influence of the social determinants of health on access to healthcare services among refugees in Australia. Aust. J. Prim. Health.

[17] Rahman M., Shindaini A., Abdullah A. (2023). Provision of education to Rohingya refugee children in Bangladesh: Exploring the forms of discrimination and intersectionality. Asia Pac. Educ. Rev..

[18] United Nations Refugee Agency Global Trends Report. https://www.unhcr.org/au/global-trends.

[19] United Nations Refugee Agency (2023). Mid Year Trends. https://www.unhcr.org/sites/default/files/2023-10/Mid-year-trends-2023.pdf.

[20] Hahn K., Steinhäuser J., Wilfling D., Goetz K. (2019). Quality of health care for refugees—A systematic review. BMC Int. Health Hum. Rights.

[21] Hathaway J. (2021). The Rights of Refugees Under International Law.

[22] Mahajan S., Meyer S.B. (2019). Social and structural factors that influence refugee women’s use of mental health care services in Canada: A narrative review. J. Health Soc. Sci..

[23] Lebano A., Hamed S., Bradby H., Gil-Salmerón A., Durá-Ferrandis E., Garcés-Ferrer J., Azzedine F., Riza E., Karnaki P., Zota D. (2020). Migrants’ and refugees’ health status and healthcare in Europe: A scoping literature review. BMC Public Health.

[24] Robertshaw L., Dhesi S., Jones L. (2017). Challenges and facilitators for health professionals providing primary healthcare for refugees and asylum seekers in high-income countries: A systematic review and thematic synthesis of qualitative research. BMJ Open.

[25] Darebo T., Spigt M., Teklewold B., Badacho A., Mayer N., Teklewold M. (2024). The sexual and reproductive healthcare challenges when dealing with female migrants and refugees in low and middle-income countries (a qualitative evidence synthesis). BMC Public Health.

[26] Kanengoni-Nyatara B., Watson K., Galindo C., Charania N., Mpofu C., Holroyd E. (2024). Barriers to and recommendations for equitable access to healthcare for migrants and refugees in Aotearoa, New Zealand: An integrative review. J. Immigr. Minor. Health.

[27] Naffah C. Syrian Refugees in Lebanon: Legal Challenges for Municipalities. https://www.lcps-lebanon.org/en/articles/details/4884/syrian-refugees-in-lebanon-legal-challenges-for-municipalities.

[28] Coumans J., Wark S. (2024). A scoping review on the barriers to and facilitators of health services utilisation related to refugee settlement in regional or rural areas of the host country. BMC Public Health.

[29] Hewitt T., Cook N. (2023). Bridging infrastructure: Conceptualising non-state organisations in complex refugee settlement service landscapes. Geoforum.

[30] Nguyen T.P., Al Asaad M., Sena M., Slewa-Younan S. (2024). Loneliness and social isolation amongst refugees resettled in high-income countries: A systematic review. Soc. Sci. Med..

[31] Lim M., Van Hulst A., Pisanu S., Merry L. (2022). Social isolation, loneliness and health: A descriptive study of the experiences of migrant mothers with young children (0–5 years old) at la maison bleue. Front. Glob. Women’s Health.

[32] Giglitto D., Ciolfi L., Bosswick W. (2022). Building a bridge: Opportunities and challenges for intangible cultural heritage at the intersection of institutions, civic society, and migrant communities. Int. J. Herit. Stud..

[33] Eggerman J., Dajani R., Kumar P., Chui S., Qtaishat L., El Kharouf A., Panter-Brick C. (2023). Social networks, empowerment, and wellbeing among Syrian refugee and Jordanian women: Implications for development and social inclusion. World Dev..

[34] Feinberg I., O’Connor M.H., Owen-Smith A., Dube S.R. (2021). Public health crisis in the refugee community: Little change in social determinants of health preserve health disparities. Health Educ. Res..

[35] Kemmak A., Nargesi S., Saniee N. (2021). Social determinant of mental health in immigrants and refugees: A systematic review. Med. J. Islam. Repub. Iran.

[36] Abi Chahine M., Kienzler H. (2022). Ageism, an invisible social determinant of health for older Syrian refugees in Lebanon: A service providers’ perspective. Confl. Health.

[37] Chaudhry A., Hebert-Beirne J., Alessi E.J., Khuzam M.Z., Mitchell U., Molina Y., Wasfie D., Fox S., Abboud S. (2024). Exploring the Health Impact of Intersectional Minority Identity Stressors on Arab Sexual Minority Women Migrants to the United States. Qual. Health Res..

[38] Yacob-Haliso O. (2016). Intersectionality and durable solutions for refugee women in Africa. J. Peacebuilding Dev..

[39] Ortlieb R., Baumgartner P., Palinkas M., Eggenhofer-Rehart P., Ressi E. (2024). Employment outcomes of refugee women and men: Multiple gender gaps and the importance of high-skill jobs. J. Ethn. Migr. Stud..

[40] Ivanova O., Rai M., Kemigisha E. (2018). A systematic review of sexual and reproductive health knowledge, experiences and access to services among refugee, migrant and displaced girls and young women in Africa. Int. J. Environ. Res. Public Health.

[41] Shishehgar S., Gholizadeh L., DiGiacomo M., Green A., Davidson P. (2017). Health and socio-cultural experiences of refugee women: An integrative review. J. Immigr. Minor. Health.

[42] Chahine M., Kirkwood M., Al-Anani A., MacKenzie-Ede F., Charbonneau K., Metersky K. (2024). Health and well-being of refugee women in sex work: A systematic literature review. Int. Health Trends Perspect..

[43] ISS Blog Creating a space for Congolese to talk about issues including how widespread sexual abuse is ravaging the Democratic Republic of the Congo’s humanitarian sector. https://issblog.nl/2023/11/23/humanitarian-observatories-series-creating-a-space-for-congolese-to-talk-about-issues-including-how-widespread-sexual-abuse-is-ravaging-the-democratic-republic-of-the-congos-humanitarian-se/.

[44] Ishaya B.J., Paraskevadakis D., Bury A., Bryde D. (2024). A systematic literature review of modern slavery through benchmarking global supply chain. Benchmarking Int. J..

[45] Srihari G., Ishaan S., Yasmina B., Florencio V.C., Victoria K., Aryan S., Aidan L., Celine H. (2024). Climate Change, Modern Slavery, and its Impact on Health—A Youth Perspective and Global Call to Action. J. Clim. Change Health.

[46] Pertek S., Block K., Goodson L., Hassan P., Hourani J., Phillimore J. (2023). Gender-based violence, religion and forced displacement: Protective and risk factors. Front. Hum. Dyn..

[47] United Nations Refugee Agency (2021). UNHCR urges support to address worsening gender-based violence impact on displaced women and girls. https://www.unhcr.org/search?search=gender-based%20violence%20impact%20on%20displaced%20women%20and%20girls%202021&sm_site_name[]=Global%20site.

[48] Phillimore J., Block K., Bradby H., Ozcurumez S., Papoutsi A. (2023). Forced migration, sexual and gender-based violence and integration: Effects, risks and protective factors. J. Int. Migr. Integr..

[49] Muhaidat N., Karmi J., Karam A., Abushaikha F., Alshrouf M. (2024). Period poverty, reuse needs, and depressive symptoms among refugee menstruators in Jordan’s camps: A cross-sectional study. BMC Women’s Health.

[50] World Health Organisation Disability. https://www.who.int/health-topics/disability#tab=tab_1.

[51] Hossain M., Pearson R., McAlpine A., Bacchus L., Muuo S.W., Muthuri S.K., Spangaro J., Kuper H., Franchi G., Cordero R.P. (2020). Disability, violence, and mental health among Somali refugee women in a humanitarian setting. Glob. Ment. Health.

[52] Tanabe M., Nagujjah Y., Rimal N., Bukania F., Krause S. (2015). Intersecting sexual and reproductive health and disability in humanitarian settings: Risks, needs, and capacities of refugees with disabilities in Kenya, Nepal, and Uganda. Sex. Disabil..

[53] Scheer S., Mondaca M. (2022). Unseen abilities–how refugee women with disabilities experience social participation. Eur. J. Public Health.

[54] United Nations Refugee Agency Integration Handbook for Resettled Refugees. https://www.unhcr.org/handbooks/ih/.

[55] World Health Organisation Global Report on Health Equity for Persons with Disabilities. https://www.who.int/teams/noncommunicable-diseases/sensory-functions-disability-and-rehabilitation/global-report-on-health-equity-for-persons-with-disabilities.

[56] Strong J., Varady C., Chahda N., Doocy S., Burnham G. (2015). Health status and health needs of older refugees from Syria in Lebanon. Confl. Health.

[57] Frost C., Morgan N., Allkhenfr H., Dearden S., Ess R., Albalawi W., Berri A., Benson L., Gren L. (2019). Determining physical and mental health conditions present in older adult refugees: A mini-review. Gerontology.

[58] Ekoh P., Walsh C. (2024). Valuable beyond vulnerable: A scoping review on the contributions of older forced migrants in post-migration recovery. Int. J. Disaster Risk Sci..

[59] Davis L. (2024). Intersectionality and Mental Health Outcomes among LGBTQ+ Refugees in United States. Eur. J. Gend. Stud..

[60] Powell A. (2024). The place where only gays go: Constructions of queer space in the narratives of sexually diverse refugees. J. Place Manag. Dev..

[61] Held N. (2023). “As queer refugees, we are out of category, we do not belong to one, or the other”: LGBTIQ+ refugees’ experiences in “ambivalent” queer spaces. Ethn. Racial Stud..

[62] Shaw A., Verghese N. (2022). LGBTQI+ refugees and asylum seekers. https://escholarship.org/uc/item/1fc1v573.

[63] Dawson A., Adjei-Mensah E., Hayen A., Nathan S., Heywood A., Mahimbo A., Merrington H., Rogers C. (2025). Health assets among refugees in Australia: A systematic review. BMC Public Health.

[64] Davidson N., Hammarberg K., Romero L., Fisher J. (2022). Access to preventive sexual and reproductive health care for women from refugee-like backgrounds: A systematic review. BMC Public Health.

[65] Jallow M., Haith-Cooper M., Hargan J., Balaam M.-C. (2022). A systematic review to identify key elements of effective public health interventions that address barriers to health services for refugees. J. Public Health.

[66] Statista Opinion on Refugees Worldwide in 2023, by Statement. https://www.statista.com/statistics/1364740/opinion-on-refugee-global-average/.

[67] Postelnicescu C. (2016). Europe’s new identity: The refugee crisis and the rise of nationalism. Eur. J. Psychol..

[68] Secen S., Al S., Arslan B. (2024). Electoral dynamics, new nationalisms, and party positions on Syrian refugees in Turkey. Turk. Stud..

[69] Joppke C. (2024). Neoliberal nationalism and immigration policy. J. Ethn. Migr. Stud..

[70] Groutsis D., Collins J., Reid C. (2024). “I’m Not a Refugee Girl, Call Me Bella”: Professional Refugee Women, Agency, Recognition, and Emancipation. Bus. Soc..

[71] Women for Women What Refugee Women Face. https://www.womenforwomen.org/blogs/5-facts-about-what-refugee-women-face.

[72] Newman A., Macaulay L., Dunwoodie K. (2023). Refugee entrepreneurship: A systematic review of prior research and agenda for future research. Int. Migr. Rev..

[73] McQuaid J.H., Mandavia A., Cassidy G., Silva M.A., Esmail K., Aragula S., Gamez G., McKenzie K. (2024). Persecution as stigma-driven trauma: Social determinants, stigma, and violence in asylum seekers in the United States. Soc. Sci. Med..

[74] Jamal Z., ElKhatib Z., AlBaik S., Horino M., Waleed M., Fawaz F., Loffreda G., Seita A., Witter S., Diaconu K. (2022). Social determinants and mental health needs of Palestine refugees and UNRWA responses in Gaza during the COVID-19 pandemic: A qualitative assessment. BMC Public Health.

[75] Fiske L., Giotis C. (2021). Refugees, Gender and Disability: Examining Intersections Through Refugee Journeys. The Palgrave Handbook of Gender and Migration.

[76] Diab J.L., Samneh B. (2024). On the margins of refuge: Queer Syrian refugees and the politics of belonging and mobility in post-2019 Lebanon. Int. J. Discrim. Law.

[77] Ekoh P.C., Iwuagwu A.O., George E.O., Walsh C.A. (2023). Forced migration-induced diminished social networks and support, and its impact on the emotional wellbeing of older refugees in Western countries: A scoping review. Arch. Gerontol. Geriatr..

[78] Piotrowicz K., Semeniv S., Kupis R., Ryś M., Perera I., Gryglewska B., Gąsowski J. (2022). Disease burden in older Ukrainian refugees of war: A synthetic reanalysis of public records data. Lancet Healthy Longev..

[79] Petersen M.J., Larsen S., Tan N.F. (2025). ‘You can never feel safe’: Danish revocation practice and the production of radical uncertainty. J. Ethn. Migr. Stud..

[80] Gammeltoft-Hansen T. (2018). The normative impact of the global compact on refugees. Int. J. Refug. Law.

[81] McAdam J., Wood T. (2021). The Concept of “International Protection” in the Global Compacts on Refugees and Migration. Interventions.

[82] Pérez-Sánchez M., Immordino P., Romano G., Giordano A., García-Gil C., Morales F. (2024). Access of migrant women to sexual and reproductive health services: A systematic. Midwifery.

[83] Marcu S. (2018). Refugee students in Spain: The role of universities as sustainable actors in institutional integration. Sustainability.

[84] Abamosa J. (2023). Social inclusion of refugees into higher education: Policies and practices of universities in Norway. Educ. Rev..

[85] Clarke S., Toole-Anstey C., Cameron J., Spence N., Spangaro J. (2024). A Rapid Evidence Review of Interventions to Identify, Prevent, and Address Intimate Partner Violence Experienced by Refugee Women in Post-Settlement Settings. Violence Gend..

[86] Finnegan A. (2022). Growing up white saviors. J. Appl. Soc. Sci..

[87] Flavel J., Due C., Howe J., Ziersch A. (2024). Refugee women and work: Evidence from an Australian longitudinal study. International Migration.

[88] O’Mahony J., Kassam S., Clark N., Asbjoern T. (2023). Use of participatory action research to support Syrian refugee mothers in the resettlement period in Canada: A longitudinal study. PLoS ONE.

[89] Flanagan N., Travers A., Vallières F., Hansen M., Halpin R., Sheaf G., Rottmann N., Johnsen A. (2020). Crossing borders: A systematic review identifying potential mechanisms of intergenerational trauma transmission in asylum-seeking and refugee families. Eur. J. Psychotraumatol..

[90] Larrea-Schiavon S., Vázquez-Quesada L.M., Bartlett L.R., Lam-Cervantes N., Sripad P., Vieitez I., Coutiño-Escamilla L. (2022). Interventions to improve the reproductive health of undocumented female migrants and refugees in protracted situations: A systematic review. Glob. Health: Sci. Pract..

[91] Ajjarapu A., Story W.T., Haugsdal M. (2021). Addressing obstetric health disparities among refugee populations: Training the next generation of culturally humble OB/GYN medical providers. Teach. Learn. Med..

